# Selpercatinib as First-Line Therapy in a 9-Month-Old Infant With Metastatic *RET* Fusion–Positive Sarcoma: A Case Report

**DOI:** 10.1200/PO-26-00232

**Published:** 2026-06-03

**Authors:** Anna-Sophia Mast, Ursula Leuschner, Stefanie Schulte, Markus Mezger, Martin Ebinger

**Affiliations:** ^1^ Department of Hematology, Oncology, Gastroenterology, Nephrology, Rheumatology, University Children's Hospital Tübingen, Tübingen, Germany; ^2^ German Cancer Consortium (DKTK) and German Cancer Research Center (DKFZ), Partner Site Tübingen, Tübingen, Germany

## Background

Infantile fibrosarcoma (IFS) is the most common soft tissue sarcoma in infants younger than 1 year. Standard treatment includes surgical resection when feasible or vincristine-/actinomycin D–based chemotherapy for unresectable tumors, achieving long-term survival rates above 90%.^1^ Next-generation sequencing (NGS) has identified *NTRK* gene fusions as a defining feature of IFS, enabling targeted therapy with TRK inhibitors, which have demonstrated rapid and durable responses, particularly in refractory or metastatic disease.^2^ Tumors with similar histopathology but lacking *NTRK* fusions are classified as IFS-like sarcomas, a heterogeneous group that may harbor alternative gene rearrangements, including *RET* fusions.^3^

*RET* fusions are most commonly observed in non–small cell lung cancers (NSCLCs, 1%-2%) and papillary thyroid carcinomas (5%-10%), but have also been reported in several other malignancies, whereas activating *RET* mutations are commonly observed in medullary thyroid cancer.^4^ Selpercatinib, a highly selective, ATP-competitive RET kinase inhibitor, has demonstrated clinical efficacy across *RET*-rearranged malignancies. In the LIBRETTO-001 trial, it achieved durable responses in both treatment-naïve and pretreated patients, with phase III studies confirming superior outcomes over standard therapies in *RET*-driven NSCLC and thyroid cancer.^5-9^ Pediatric efficacy was demonstrated in the LIBRETTO-121 and Pediatric MATCH trials, with response rates of up to 83% and the 2-year progression-free survival exceeding 90%.^10,11^ Since 2024, selpercatinib has been approved by the US Food and Drug Administration as a tumor-agnostic therapy for pediatric patients older than 2 years, with advanced *RET* fusion–positive solid tumors lacking satisfactory treatment alternatives.^12^

To our knowledge, we present the first documented use of selpercatinib as first-line therapy in an infant younger than 1 year diagnosed with metastatic IFS-like sarcoma harboring a *VCL*::*RET* gene fusion. Treatment was initiated at 9 months of age and resulted in rapid and sustained tumor regression. After 1 year of therapy, the primary lesion had regressed to a desmoplastic scar and pulmonary metastases had completely resolved. Selpercatinib was well-tolerated, with only grade 1-2 adverse events and no requirement for dose modification. This case highlights the feasibility and therapeutic potential of *RET*-targeted therapy in infants and underscores the importance of early molecular profiling in pediatric sarcomas.

## Initial Case Presentation

**FIG 1.**
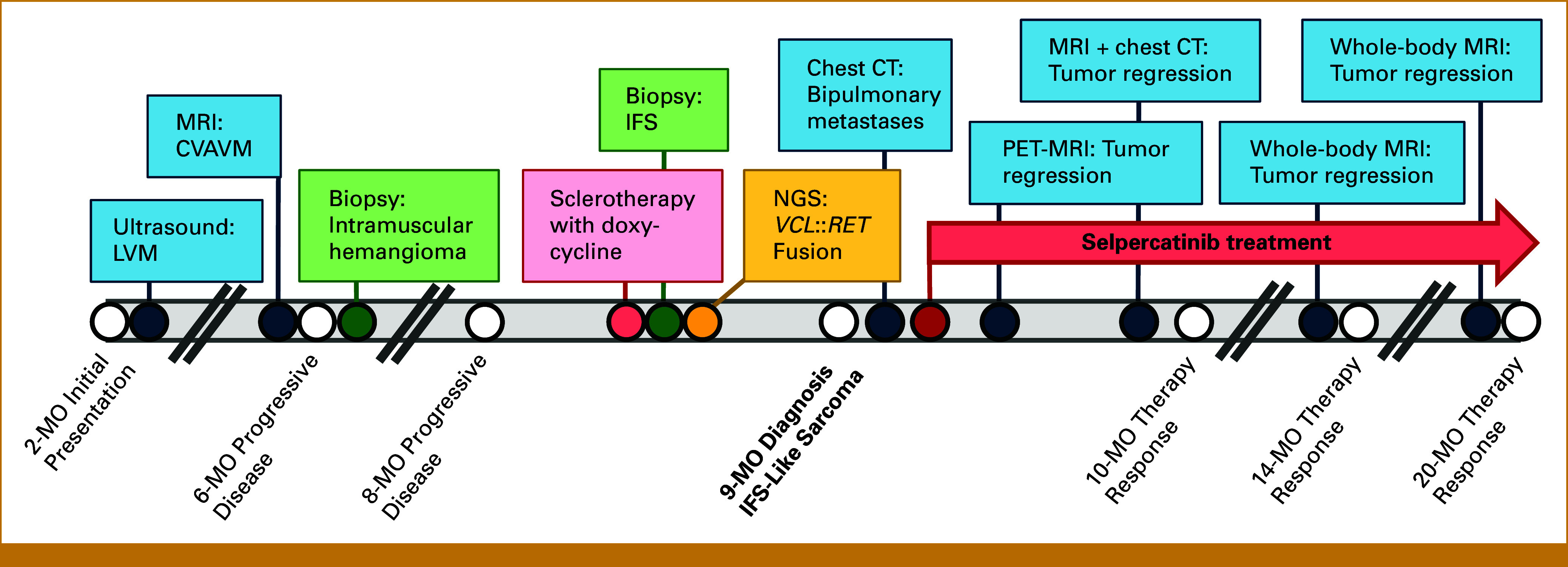
Clinical course of an infant with *RET* fusion–positive IFS-like sarcoma treated with selpercatinib. The schematic illustrates the clinical course from initial presentation at 2 months of age through diagnostic evaluation, treatment, and response assessment. Key diagnostic and therapeutic interventions are displayed in chronological order, aligned with the patient's age. Color coding denotes diagnostic modalities (blue), biopsy results (green), molecular findings (yellow), and therapeutic interventions (red). CT, computed tomography; CVAVM, capillary-venous-arteriovenous malformation; IFS, infantile fibrosarcoma; LVM, lymphatic-venous malformation; MO, months; MRI, magnetic resonance imaging; NGS, next-generation-sequencing; PET-MRI, positron emission tomography-MRI.

At 2 months of age, the patient presented with a soft tissue mass in the proximal left thigh, initially suspected to be a lymphatic-venous malformation (LVM; Fig 1). The lesion measured 2.8 × 4.8 × 4.7 cm and demonstrated partial intramuscular involvement. Progressive growth over the following 3 months raised concern for a capillary-venous-arteriovenous malformation, prompting a biopsy. Histopathologic evaluation initially indicated a benign intramuscular hemangioma. However, continued tumor growth led to transfusion-dependent anemia, necessitating doxycycline sclerotherapy at 8 months and prompting a second biopsy. This revealed a spindle cell tumor with strong S100 expression and a Ki-67 proliferation index of up to 60%, with extended immunohistochemical workup not supporting a vascular neoplasm and instead favoring a mesenchymal tumor consistent with IFS. While fluorescence in situ hybridization showed no *NTRK* rearrangements, targeted RNA-based NGS (Archer FusionPlex Pan Solid v2, ArcherDX, Boulder, CO) identified an in-frame *VCL*(18)::*RET*(12) gene fusion (Data Supplement, Fig S1). Based on the combined histopathologic, immunohistochemical, and molecular findings, the tumor was classified as a *RET* fusion–positive IFS-like sarcoma.

## First-Line Treatment With Selpercatinib

**FIG 2.**
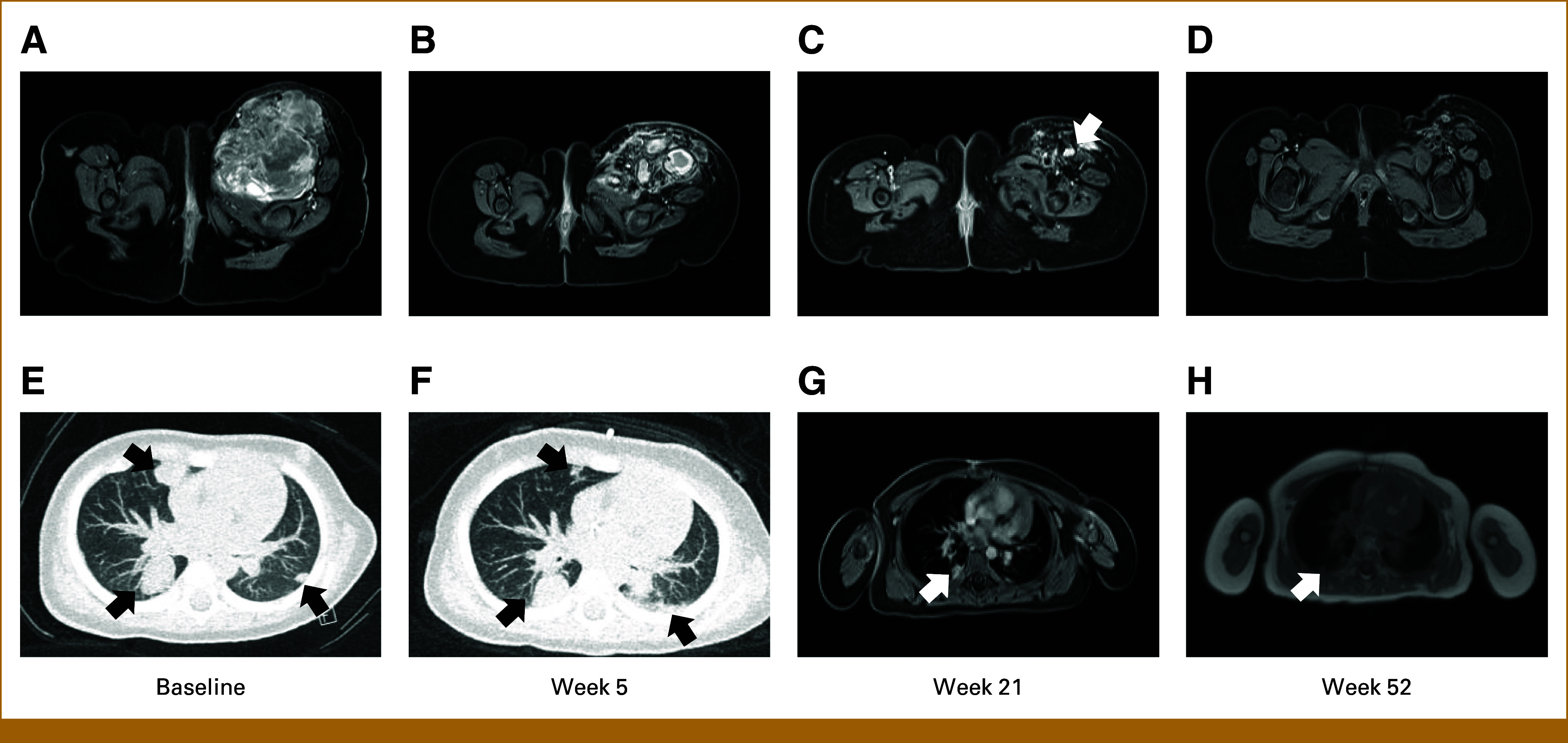
Radiographic response of primary tumor and pulmonary metastases to selpercatinib. Axial T1-weighted MRI images of the proximal left thigh at (A) baseline, (B) week 5, (C) week 21, and (D) week 52, demonstrating marked regression of the primary tumor. By week 21, one of three residual nodules is shown as a representative example (white arrow, C). The pulmonary response: (E and F) axial chest CT scans at baseline and week 5, (G) axial T1-weighted MRI at week 21, and (H) flow-sensitive 3D-MRI at week 52. Pulmonary metastases are indicated by black arrows; fibrotic remodeling at prior metastatic sites is indicated by white arrows. 3D-MRI, three-dimensional magnetic resonance imaging; CT, computed tomography; Flow-3D-MRI, flow-sensitive three-dimensional magnetic resonance imaging; MRI, magnetic resonance imaging; T1-weighted, T1-weighted imaging.

Baseline staging with whole-body magnetic resonance imaging (MRI) and chest computed tomography (CT) confirmed the primary tumor in the proximal left thigh (7.5 × 8 × 12 cm; 125 cm^3^), bilateral pulmonary metastases (largest measuring 25 × 28 mm), and a metastatic lesion in the left iliac lymph node (14 × 23 mm; Figs 2A and 2E). The disease was classified as T2bN1M1 and stage IV according to the Intergroup Rhabdomyosarcoma Study.^13^ Given the pulmonary and lymphatic metastases, surgical resection was not feasible. Based on the identified *RET* fusion, first-line treatment with selpercatinib was recommended by the institutional tumor board and the national Cooperative Weichteilsarkom Studiengruppe. After comprehensive counseling, informed consent for off-label use was obtained from both parents. Selpercatinib was initiated at 9 months of age, administered orally in continuous 28-day cycles, starting at 40 mg once daily and escalated to 40 mg twice daily (92 mg/m^2^ twice a day) on day 6, in alignment with the LIBRETTO-121 protocol.^10^

## Response to Selpercatinib

After 7 days of treatment, positron emission tomography-MRI demonstrated a marked reduction in bilateral pulmonary metastases and no detectable metabolic activity in the primary tumor or lymph node metastasis although interpretation was limited by the absence of baseline PET imaging. At 5 weeks, MRI and chest CT confirmed a continued response, with >70% reduction of the primary tumor (5.0 × 5.5 × 7.5 cm), decreased lymph node size, and substantial regression of pulmonary metastases, except for a single residual lesion in the right lower lobe (Figs 2B and 2F). By week 21, only three small nodules remained at the primary site, with a total tumor volume of 9.5 cm^3^, representing a reduction of more than 90% from baseline (Fig 2C). Pulmonary lesions had nearly completely resolved, with only pleura-based changes and fibrotic remodeling evident at the site of the previously largest lesion, now measuring 1.0 × 0.7 cm (Fig 2G). After 1 year of therapy, imaging demonstrated a desmoplastic scar with streak-like residual contrast enhancement at the primary site, consistent with a sustained treatment response (Fig 2D). MRI showed no definitive evidence of residual pulmonary metastases, with only a small, stable scar-like remnant remaining (Fig 2H). In addition, the left inguinal lymph node had further decreased in size, measuring 4 mm in axial diameter after 1 year. At the time of data cutoff, the patient remains on selpercatinib therapy.

**FIG 3.**
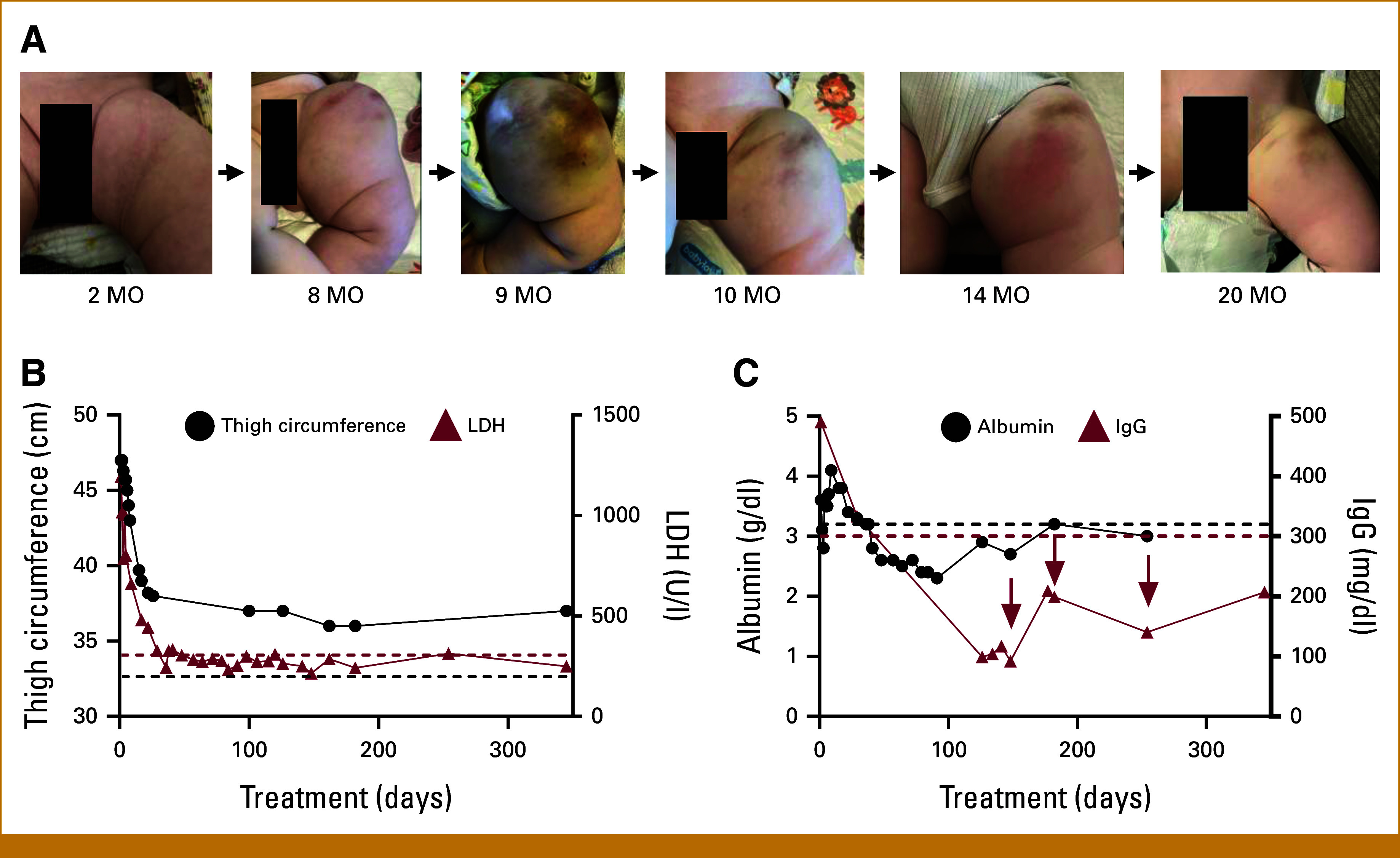
Clinical response and treatment-associated adverse events during selpercatinib therapy. (A) Clinical photographs of the primary tumor at the proximal left thigh, obtained at age 2, 8, 9, 10, 14, and 20 months (left to right), demonstrating progressive tumor enlargement followed by marked regression after initiation of selpercatinib. (B) The clinical response during the first year of selpercatinib treatment, assessed by serial measurements of thigh circumference of the affected limb (black circles, left *y*-axis) and serum LDH (red triangles, right *y*-axis). The black horizontal dashed line denotes the thigh circumference of the unaffected (right) limb at age 20 months (1 year after treatment initiation). The red horizontal dashed line indicates the upper limit of the normal range for LDH. (C) Laboratory parameters associated with treatment-related adverse events. Serum albumin (black circles, left *y*-axis) and IgG (red triangles, right *y*-axis) levels are shown throughout the course of therapy. The black and red horizontal dashed lines indicate the lower limits of the normal ranges for albumin and IgG, respectively. Arrows indicate the timing of IgG infusion. IgG, immunoglobulin G; LDH, lactate dehydrogenase; MO, months.

Clinically, the circumference of the affected limb decreased from 47.0 cm at baseline to 37.0 cm after 1 year, with the first reduction observed as early as day 3 (Figs 3A and 3B). The contralateral, unaffected limb measured 28.0 cm at baseline and increased to 32.5 cm over the same period. Lactate dehydrogenase levels, initially elevated at diagnosis, peaked on day 2 (1193 U/L) and normalized by day 48, correlating with clinical and radiologic response (Fig 3B).

## Adverse Events

Throughout 1 year of selpercatinib therapy, the patient experienced grade 1 and 2 adverse events, as defined by the Common Terminology Criteria for Adverse Events version 5.0 (Data Supplement, Table S1).^14^ No events required dose modification, and treatment is ongoing. A decline in serum immunoglobulin G (IgG) levels was observed (nadir 92 mg/dL; normal range, 300-1,000 mg/dL), a finding not previously reported with selpercatinib and of unclear clinical significance (Fig 3C). Despite the hypogammaglobulinemia, the patient exhibited no clinical signs of increased susceptibility to infections. Lymphocyte subset analyses were within normal limits, and no renal IgG loss was detected. After three intravenous immunoglobulin infusions, IgG levels improved but remained below normal and monitoring is ongoing. On day 49, the patient developed grade 2 hypoalbuminemia (nadir 2.3 g/dL) with periorbital edema, which resolved spontaneously by day 195 (Fig 3C). Additional adverse events included grade 1 fever at treatment initiation, transient asymptomatic sinus bradycardia after dose escalation on day 6, and localized eczema at the site of the Hickman catheter.

## Informed Consent

Written informed consent was obtained from the patient's parents for treatment and for publication of this case report and accompanying images.

## Ethical Approval

This case report was conducted in accordance with institutional guidelines. Ethical approval was not required for single-case descriptions based on retrospective clinical data.

## Discussion

To our knowledge, this case represents the first documented use of selpercatinib as first-line therapy in an infant younger than 1 year with metastatic *RET* fusion–positive IFS-like sarcoma, resulting in a rapid and sustained clinical response. The diagnosis was delayed by 7 months because of initial imaging findings suggestive of a benign vascular malformation, a known diagnostic challenge in vascularized or hemorrhagic soft tissue tumors.^15,16^ A nondiagnostic initial biopsy and continued disease progression prompted repeat tissue sampling and molecular profiling, which identified a *RET* fusion, underscoring the critical role of early genomic evaluation in pediatric malignancies.

Historically, IFS management has relied on surgery, often with significant morbidity.^17^ Vincristine-/actinomycin D–based chemotherapy later enabled organ preservation but is associated with long-term toxicities, including neuropathy, hepatotoxicity, and, rarely, secondary malignancies, particularly concerning for infants.^1,18,19^ In this case, given the metastatic and unresectable tumor and emerging evidence for *RET*-targeted therapy, selpercatinib was initiated as off-label first-line treatment.

The patient demonstrated rapid and sustained clinical improvement, consistent with interim LIBRETTO-121 data reporting a 100% response rate and a 24-month duration of response in patients with measurable disease at treatment initiation.^10^ In several case reports, similar outcomes have been reported, including a 21-month-old child with metastatic, chemotherapy-resistant IFS-like sarcoma harboring a *SPECC1L*::*RET* fusion, who exhibited significant tumor regression after selpercatinib treatment.^20^

Selpercatinib was well-tolerated, with only mild (grade 1-2) adverse events, consistent with its established safety profile and high *RET* selectivity.^5,10^ Although long-term pediatric data remain limited, adult experience from LIBRETTO-001 (median treatment >3 years) provides preliminary reassurance.^5^

In accordance with current regulatory guidance, selpercatinib is planned to be continued until disease progression or unacceptable toxicity as no defined treatment duration has been established. At the time of data cutoff, the patient remains on selpercatinib therapy with no evidence of active disease and sustained favorable tolerability. The optimal duration of therapy and the feasibility of treatment discontinuation remain unresolved challenges. In the absence of selpercatinib-specific data, experience from other targeted therapies, such as larotrectinib, suggests that elective discontinuation with close surveillance may be feasible in selected patients, although associated with a risk of relapse.^21^ Accordingly, after a prolonged period of sustained remission (eg, 2-3 years), surgical resection of any residual tumor tissue may be considered, followed by a carefully monitored watch-and-wait strategy. However, this approach remains investigational and requires further study.

This case highlights the potential of selpercatinib as a first-line treatment option for infants with *RET* fusion–positive IFS-like sarcoma, particularly when standard therapies are limited or highly morbid. In the absence of established guidelines, our findings emphasize the importance of early, comprehensive molecular profiling to guide targeted therapeutic decisions. Although long-term outcomes are yet to be determined, this report contributes to the growing evidence supporting precision oncology in rare pediatric sarcomas.

## Data Availability

The data analyzed during the case report are not publicly available because they contain individual patient information, but are available from the corresponding author on reasonable request.
